# Risk of non-Hodgkin lymphoma in breast cancer survivors: a nationwide cohort study

**DOI:** 10.1038/s41408-021-00595-0

**Published:** 2021-12-14

**Authors:** Danbee Kang, Sang Eun Yoon, Dongwook Shin, Jin Lee, Yun Soo Hong, Se Kyung Lee, Jeong Eon Lee, Yeon Hee Park, Jin Seok Ahn, Eliseo Guallar, Won Seog Kim, Jungho Lee, Seok Jin Kim, Juhee Cho

**Affiliations:** 1 Department of Clinical Research Design and Evaluation, SAIHST, Sungkyunkwan University, Seoul, Korea; 2 Center for Clinical Epidemiology, Samsung Medical Center, Sungkyunkwan University, Seoul, Korea; 3 Division of Hematology/Oncology, Department of Medicine, Samsung Medical Center, Sungkyunkwan University School of Medicine, Seoul, South Korea; 4 Department of Family Medicine, Samsung Medical Center, Sungkyunkwan University School of Medicine, Seoul, South Korea; 5 Department of Epidemiology and Medicine, and Welch Center for Prevention, Epidemiology and Clinical Research, Johns Hopkins University Bloomberg School of Public Health, Baltimore, MD USA; 6 Department of Surgery, Samsung Medical Center, Sungkyunkwan University School of Medicine, Seoul, Korea; 7 Department of Health Sciences and Technology, SAIHST, Sungkyunkwan University, Seoul, Korea; 8 Department of Plastic Surgery, The Catholic University of Korea, Bucheon ST. Mary’s Hospital, Bucheon, South Korea

**Keywords:** Risk factors, Cancer epidemiology

## Abstract

Several studies have suggested that estrogens have a protective function against lymphomagenesis. The treatment of breast cancer is driven by subtype classification, and the assessment of hormone receptor status is important for treatment selection. Thus, we evaluated the association between breast cancer and the incidence of NHL. We conducted a retrospective cohort study using a population-based nationwide registry in South Korea. We selected all women with newly diagnosed breast cancer between January 1st, 2002 and December 31st, 2016 who received curative treatment (*N* = 84,969) and a 1:10 sample of age-matched non-breast cancer controls (*N* = 1,057,674). Incident breast cancer (time-varying exposure) was the exposure and development of any type of NHL, including diffuse large B-cell lymphoma (DLBCL), follicular lymphoma (FL), mature T/NK-cell lymphomas, anaplastic large cell lymphoma (ALCL), and unspecified types of NHL, was the outcome. During follow-up, 1564 incident cases of NHL occurred. The fully adjusted Hazard Ratio (HR) for NHL associated with the development of breast cancer was 1.64 (95% CI = 1.34–2.00) after adjusting for body mass index, alcohol intake, physical activity, smoking, income, and comorbidity. The adjusted HR for NHL was much higher in participants who were aged <50 years and who received hormone therapy (either tamoxifen or aromatase inhibitors) than in those ≥50 years or who did not receive hormone therapy, respectively. The development of breast cancer was associated with a significantly increased risk of NHL, particularly follicular lymphoma and mature T/NK-cell lymphoma. In particular, the risk of NHL was higher in patients receiving hormone therapy and in younger patients.

## Introduction

Non-Hodgkin lymphoma (NHL) is the most common hematologic malignancy worldwide [1, 2]. The overall 5-year survival rate for people with NHL is 73%. For stage I NHL, the 5-year survival rate is more than 83%. For stage II the 5-year survival rate is close to 76% and for stage III it is more than 70%. For stage IV NHL, the 5-year survival rate is around 63%. These survival rates vary depending on the cancer’s stage and subtype [3]. Accordingly, there has been increasing interest in identifying the related risk factors to better understand NHL pathogenesis. However, there is no established etiology for NHL as this disease does not constitute a single disorder but rather a group of disorders involving heterogeneous subtypes with variable clinical behaviors and treatment outcomes. Currently, exact causal relationships have only been reported for some subtypes. For example, long-term immune suppression could induce NHL in patients receiving immunosuppressive agents after organ transplantation and those with human immunodeficiency virus infection [4, 5]. However, as these cases account for an extremely small proportion of NHL patients, the identification of people at risk of NHL remains a challenge.

An intriguing discovery in hemato-oncology is the lower incidence of lymphomas in women than that in men [6]. Diverse incidence patterns have been observed for distinct histologic subtypes of NHL. The male predominance has been reported in diffuse large B-cell lymphoma (DLBCL) as well as NHL overall [7]. Follicular lymphoma (FL) has a slight female predominance [7], but male sex was reported to be associated with poor clinical outcomes in FL [8]. Although female reproductive hormones were initially suspected to play a role in this phenomenon, the precise underlying mechanisms remain unclear [9]. Lymphomas are still not generally perceived as hormone-controlled; however, several studies have suggested that estrogens have a protective function against lymphomagenesis [9].

The treatment of breast cancer is driven by subtype classification, and the assessment of hormone receptor status is important for treatment selection. Although some reports have suggested an association between breast cancer and NHL incidence, these results were based on small numbers of NHL cases [10, 11]. Japanese patients with breast cancer have a 3.5-fold higher risk of NHL compared to the general population [12]. In the Netherlands, patients with early breast cancer had a 1.19-fold increased risk of NHL (95% confidence interval [CI] 0.61–2.08) compared to the general population [11]. A Korean multicenter study reported a standardized incidence ratio of NHL of 1.86 (95% CI 0.21–6.72) among 3444 patients with breast cancer, using the general population for comparison. However, previous studies had limitations because they provided little information about the type of cancer treatment, especially anti-hormone therapy, menopause, and lifestyle variables that could affect the occurrence of a second malignancy. Thus, we conducted a nationwide cohort study to evaluate the incidence of NHL in breast cancer survivors.

## Materials and methods

### Study population and design

We conducted a retrospective cohort study using a population-based national registry. Korea has a universal single-payer national health insurance system; the National Health Insurance Service (NHIS) maintains the national records of all covered inpatient and outpatient visits, procedures, and prescriptions. The NHIS also includes information on risk factor levels for common chronic conditions collected during routine health screening examinations supported by the national health system.

**Fig. 1:**
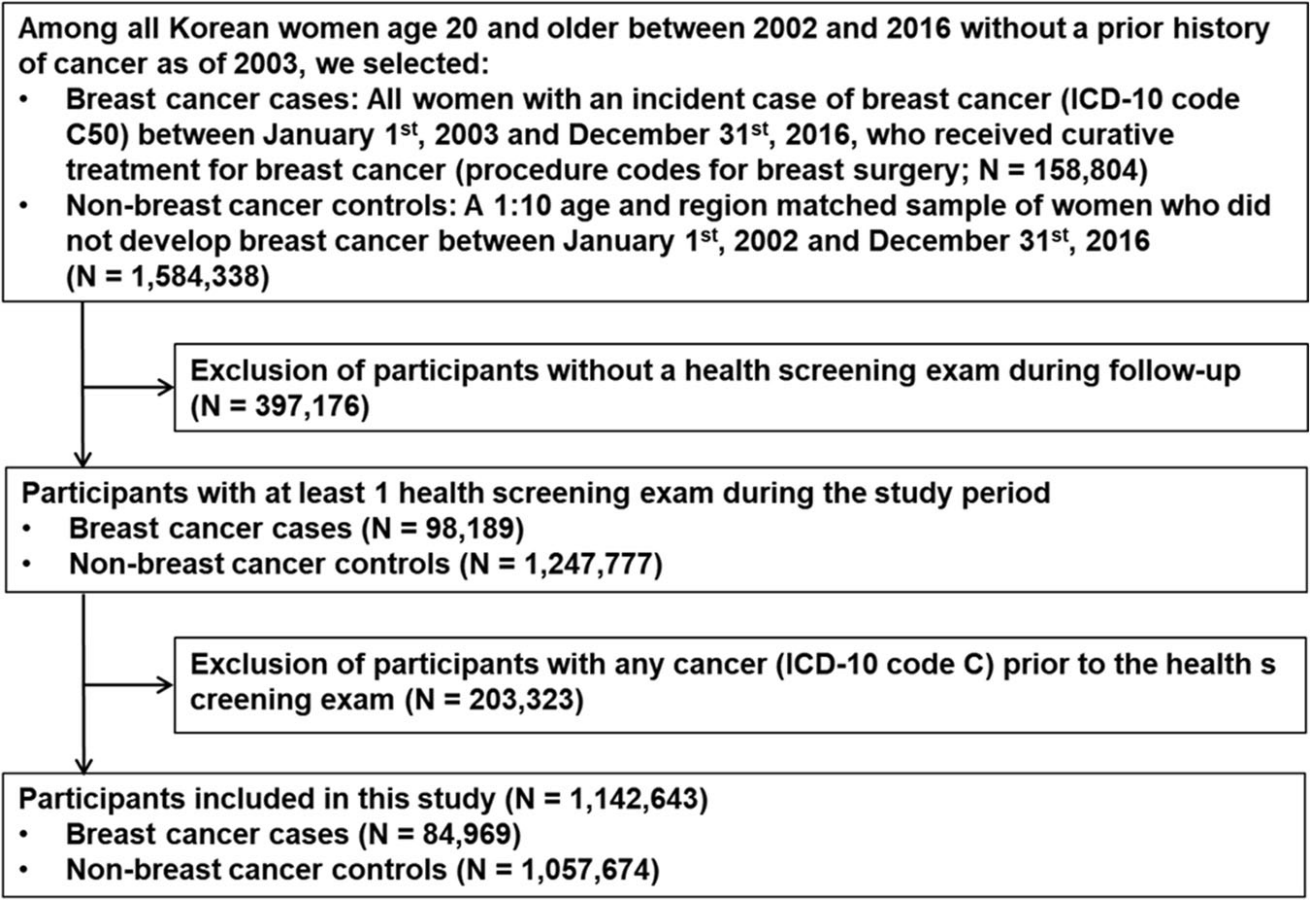
Flow chart of the study participants.

Among all Korean women aged ≥20 years between 2002 and 2016 without a history of cancer (defined as an International Classification of Diseases 10^th^ Revision [ICD-10] C code in any prior claim) as of 2002, we selected those with incident breast cancer (ICD-10 code C50) between January 1, 2003, and December 31, 2016 (Fig. 1). Because our study aimed to evaluate the long-term effects of breast cancer on the incidence of NHL, we restricted our analysis to patients who received curative treatment for breast cancer (procedure codes for breast surgery, N7131 to N7135; *N* = 158,804). We then identified a 1:10 age- and region-matched sample of women who did not develop breast cancer during the study period (non-breast cancer controls; *N* = 1,584,338). From among breast cancer cases and non-breast cancer controls, we selected participants who underwent at least one health screening exam during the study period (98,189 breast cancer cases and 1,247,777 non-breast cancer controls) and excluded 203,323 participants who presented with any cancer (ICD-10 code C) before the health screening examination, as the health screening examination constituted the baseline for the current study. The final sample size was 1,142,643 (84,969 breast cancer cases and 1,057,674 non-breast cancer controls). The Institutional Review Board of Samsung Medical Center approved this study (IRB number: 2017-08-147) and waived the requirement for informed consent, as we used only de-identified data.

### Data collection

The NHIS data comprises data from four databases: those on insurance eligibility, medical treatments, medical care institutions, and general health examinations. In addition, we used national cancer screening data. The insurance eligibility database contains information on patient age, sex, residential area, type of health insurance, income level, and disability. The medical treatment database contains information on treatment bills, including details of diseases and prescriptions. NHIS claims for inpatient and outpatient visits, procedures, and prescriptions were coded using the ICD-10, which was adopted in Korea in 1995, and the Korean Drug and Anatomical Therapeutic Chemical Codes [13, 14].

The main study exposure was the development of incident breast cancer (ICD-10 code C50). Breast cancer was confirmed based on the presence of a C50 code in more than three claims within 1 year or in an inpatient hospitalization claim in the medical treatment database. Treatments for breast cancer were identified from claims filed within 1 year of breast cancer diagnosis and used to classify patients as those treated with only surgery or surgery plus chemotherapy, radiation therapy, or hormone treatment.

The study outcome was the development of NHL (all subtypes). NHL was confirmed based on the presence of C82–C85 or C96 codes in more than three claims within 1 year or in an inpatient. We further categorized NHL into five groups: Diffuse large B-cell lymphoma (DLBCL) (ICD-10, C83.3), follicular lymphoma (ICD-10, C82), mature T/NK-cell lymphomas (ICD-10, C84), ALCL (ICD-10, C84.6, C84.7), and others (C96). The NHIS routinely audits the claims; these data are considered reliable and have been used in numerous peer-reviewed publications [1, 15]. According to a recent study, definitions of cancer diagnosis in our study is an accurate and valid method to identify cancer incidence using health insurance claim data [16].

Patient age and income levels were determined from the insurance eligibility database. Data on smoking habits, drinking habits, physical activity, height, and weight were obtained from the health screening examination database. Body mass index (BMI) was calculated as weight in kilograms divided by height in meters squared. We used data on the age at menarche and age at menopause from the national cancer screening data and generated a variable for menopausal status at the first health screening examination.

### Statistical analysis

The participants were included in the study on the date of their first health screening examination (baseline) and were followed up until the development of a study endpoint, death, or the end of the study period (December 31, 2016). The study endpoint was the development of NHL.

The study exposure was breast cancer development, which was considered a time-varying variable. Thus, unexposed person-time was contributed by women who never developed breast cancer, as well as by women who developed breast cancer before developing it. For each outcome, cases of breast cancer occurring after developing a study endpoint (outcome) were not included in the analysis as participant follow-up ended with the development of a study outcome. Since cancer patients receive various exams prior to cancer treatment, to reduce the potential impact of surveillance bias we considered that outcomes occurring in the first 31 days after a diagnosis of breast cancer corresponded to unexposed person-time. Cumulative incidences were estimated using the Kaplan–Meier method. We calculated hazard ratios (HRs) with 95% CIs for developing NHL using a proportional hazards regression model with age as the time scale.

To account for potential confounding factors at the health screening examination (baseline), we adjusted for BMI categories (underweight, normal, overweight, obese, and unknown), alcohol intake (none, moderate, heavy, and unknown), moderate–vigorous physical activity (none, 1–2 times per week, >3 times per week, and unknown), smoking status (never, ever, or unknown), income percentile (Medical Aid, ≤30^th^, 31^st^–70^th^, >70^th^ percentile), and presence of comorbid conditions at the time of the health screening examination.

To estimate age-dependent effects, we conducted time-varying analyses according to the split-age interval. Participants were assigned to the <50 years age group until they reached the age of 50 years and to the age ≥ 50 years age group. Furthermore, to evaluate the temporal effects of breast cancer, follow-up duration was classified as <1, 1 to <3, 3 to <5, and ≥5 years from the time of breast cancer diagnosis.

We also performed an additional analysis in women who developed breast cancer to estimate treatment effects. Owing to the substantial overlap of treatment modalities (chemotherapy, radiation therapy, and hormone therapy), we used non-exclusive categories in the analysis of treatment modalities (for instance, the analysis of participants who received chemotherapy compared all participants who received chemotherapy to those who did not, irrespective of other treatment modalities). In addition, to account for competing risks due to mortality, we fitted a proportional subdistribution hazards regression model with death as the competing event [17]. We examined the proportional hazards assumption using plots of the log(−log) survival function and Schoenfeld residuals. All analyses were performed using STATA version 16 (StataCorp LP, College Station, TX, USA).

## Results

**Fig. 2:**
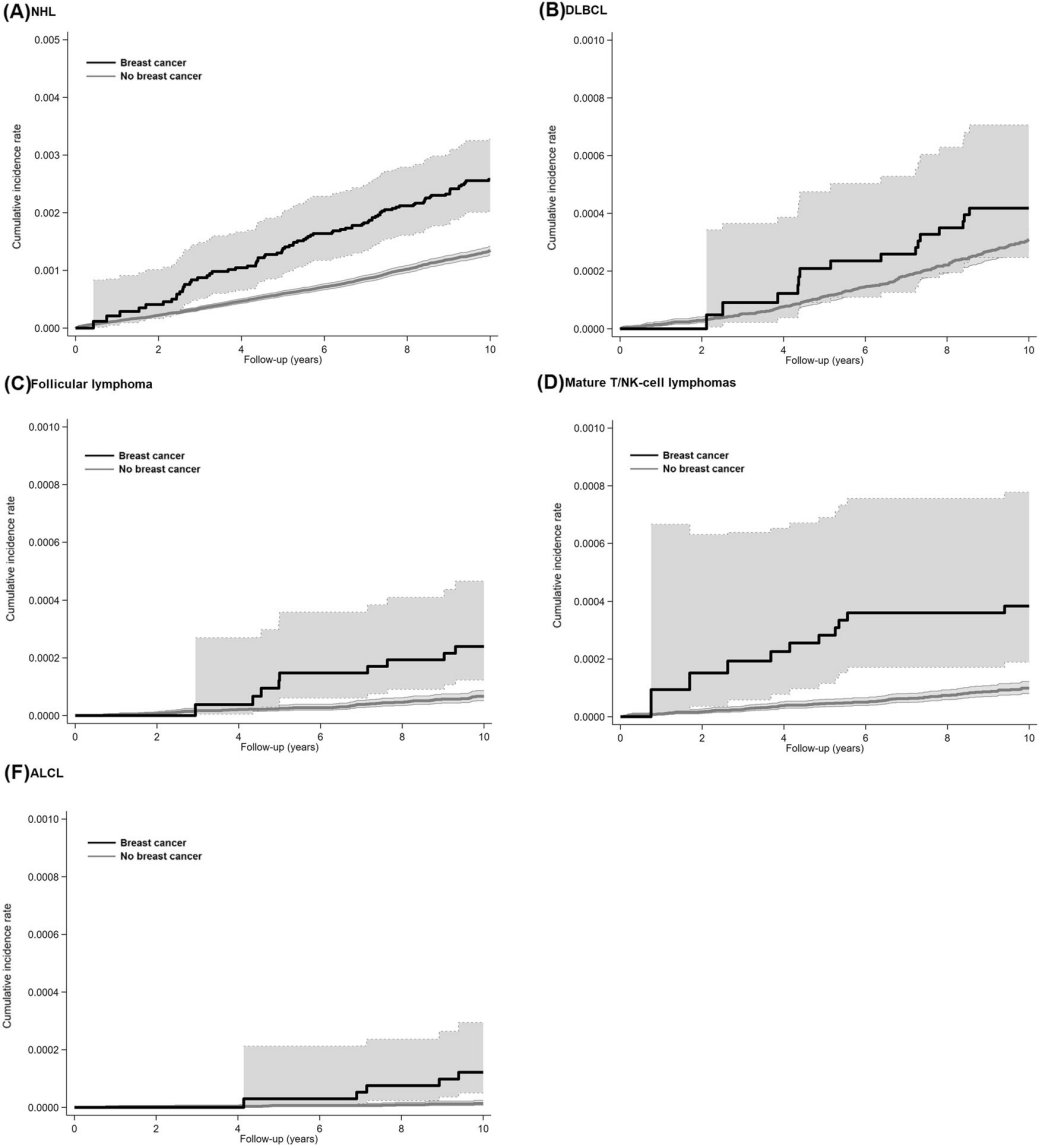
Hazard ratios and cumulative incidences of NHL by breast cancer status. **A** NHL, **B** DLBCL, **C** Follicular lymphoma, **D** Mature T/NK-cell lymphomas, and **E** ALCL. The cumulative incidences were calculated using Kaplan–Meier curves. Participants who developed breast cancer contributed person-time to the exposed group from the time of breast cancer development Unexposed person-time was contributed by participants who did not develop breast cancer and by participants who developed breast cancer prior to each comorbidity development. To reduce the potential impact of surveillance bias, we considered outcomes occurring in the first 31 days after breast cancer diagnosis as unexposed person-time. ALCL anaplastic large cell lymphoma, DLBCL Diffuse large B-cell lymphoma, NHL non-Hodgkin lymphoma.

Compared to participants who developed breast cancer (*N* = 84,969; 424,669 person-years of follow-up), non-breast cancer controls (*N* = 1,057,674; 10,648,319 person-years of follow-up) had similar characteristics at the time of the baseline health screening examination (Table 1). During 11,072,988 person-years of follow-up, 1564 incident cases of NHL occurred. Of these, 105 occurred after the development of breast cancer (incidence rate 2475 per 10,000,000 person-years), and 1459 occurred before the development of breast cancer or in participants who did not develop cancer (incidence rate 1370 per 10,000,000 person-years; Fig. 2A and Table 2). The HR for NHL associated with the development of breast cancer was 1.64 (95% CI = 1.34–2.00). The association did not change significantly after adjusting for confounding factors (fully adjusted HR = 1.64; 95% CI = 1.34–2.00). While participants with obesity and ever smokers had a higher risk of NHL, the associations were not statistically significant (Supplement Table 1). In addition, a proportional subdistribution hazards regression model with death as the competing event showed similar findings (not shown). The development of breast cancer was associated with a significantly increased risk of follicular lymphoma (HR = 3.56, 95% CI = 1.88, 6.72), mature T/NK-cell lymphoma (HR = 2.75, 95% CI = 1.55, 4.90), and ALCL (HR = 7.46; 95% CI = 2.70–20.59, Table 2).

**Table 1:** Characteristics of the study participants at the baseline health screening examination (*N* = 1,142,643).

Characteristics	No breast cancer (*N* = 1,057,674)	Incident breast cancer (*N* = 84,969)
*Age at baseline, years*	46 (40–54)	46 (40–53)
*Age at menarche, years*		
<10	2660 (0.3)	394 (0.5)
10–13	73,185 (6.9)	7025 (8.3)
14–16	482,512 (45.6)	41,278 (48.6)
≥17	398,988 (37.7)	27,696 (32.6)
Unknown	100,329 (9.5)	8578 (10.1)
*Menopause status*		
Pre-menopause	404,672 (38.3)	30,291 (35.7)
Post-menopause	556,829 (52.7)	46,501 (54.7)
Missing data	96,163 (9.1)	8177 (9.6)
BMI, kg/m^2^		
Underweight (<18)	38,847 (3.7)	3035 (3.6)
Normal (18 to <23)	474,009 (44.8)	37,724 (44.4)
Overweight (23 to <25)	243,220 (23.0)	19,577 (23.0)
Obese (≥25)	301,127 (28.5)	24,604 (29.0)
Missing data	471 (0.0)	29 (0.0)
*Alcohol consumption*		
None	713,524 (67.5)	58,034 (68.3)
Moderate (1–19 g/day)	275,882 (26.1)	21,469 (25.3)
Heavy (≥20 g/day)	20,848 (2.0)	1497 (1.8)
Missing	47,420 (4.5)	3978 (4.7)
Smoking status		
Never smoker	946,369 (89.5)	75,647 (89.0)
Ever smoker	58,619 (5.5)	4767 (5.6)
Missing data	52,686 (5.0)	4555 (5.4)
*Moderate–vigorous physical activity*		
None	610,131 (57.7)	48,480 (57.1)
1–2 times per week	204,126 (19.3)	16,719 (19.7)
≥3 times per week	195,661 (18.5)	15,701 (18.5)
Missing	47,756 (4.5)	4069 (4.8)
*Income percentile*		
Medical Aid	19,753 (1.9)	1480 (1.7)
≤30^th^	304,541 (28.8)	23,692 (27.9)
31^st^–70^th^	357,873 (33.8)	28,465 (33.5)
>70^th^	375,507 (35.5)	31,332 (36.9)

BMI body mass index.

Values in the table are medians (interquartile range) or numbers (percentage).

**Table 2:** Hazard ratios (95% CI) for incident NHL associated with incident breast cancer.

	Incidence rate per 10,000,000 person-years		Adjusted HR (95% CI)	*P*-value
	No breast cancer	Breast cancer		
NHL	1370	2473	1.64 (1.34, 2.00)	<0.01
Age < 50 years	773	2354	2.91 (2.03, 4.16)	<0.01
Age ≥ 50 years	1821	2531	1.36 (1.08, 1.73)	0.01
DLBCL	308	424	1.19 (0.74, 1.92)	0.47
Age < 50 years	124	214	1.49 (0.47, 4.76)	0.50
Age ≥ 50 years	447	527	1.15 (0.68, 1.93)	0.61
Follicular lymphoma	69	259	3.56 (1.88, 6.72)	<0.01
Age < 50 years	37	357	10.22 (3.73, 27.96)	<0.01
Age ≥ 50 years	92	211	2.26 (0.98, 5.26)	0.06
Mature T/NK-cell lymphomas	102	306	2.75 (1.55, 4.90)	<0.01
Age < 50 years	63	214	3.16 (0.96, 10.44)	0.06
Age ≥ 50 years	132	351	2.65 (1.37, 5.11)	<0.01
ALCL	14	118	7.46 (2.70, 20.59)	<0.01
Age < 50 years	7	14	18.82 (3.11, 113.70)	<0.01
Age ≥ 50 years	20	105	5.28 (1.49, 18.74)	<0.01
Other NHL*	89	148	1.54 (1.19, 1.98)	<0.01
Age < 50 years	55	157	2.78 (1.80, 4.31)	<0.01
Age ≥ 50 years	115	144	1.23 (0.90, 1.69)	0.19

HR hazard ratio, CI confidence interval, ALCL anaplastic large cell lymphoma, DLBCL Diffuse large B-cell lymphoma, NHL non-Hodgkin lymphoma.

Age as the time scale. Adjusted for body mass index categories (underweight, normal, overweight, obese, and unknown), alcohol consumption (none, moderate, heavy, and unknown), moderate–vigorous physical activity (none, 1–2 times per week, >3 times per week, and unknown), smoking status (never, ever, and unknown), and income percentile (Medicaid, ≤30th, 31st–70th, >70th).

The adjusted HR for NHL was much higher in participants aged <50 years (fully adjusted HR = HR = 2.91; 95% CI = 2.03–4.16) than in older participants (fully adjusted HR = 1.36; 95% CI = 1.08–1.73) (*P* for interaction < .01). Age-related effects were observed for all subtypes (Table 2). The excess risk for NHL was higher in the first 2 years after breast cancer diagnosis (Table 3). The fully adjusted HRs for NHL at <1, 1 to <3, 3 to <5, and ≥5 years after breast cancer diagnosis were 2.59 (95% CI = 1.79–3.74), 1.62 (95% CI = 1.13–2.31), 1.41 (95% CI = 0.91–2.19), and 1.29 (95% CI = 0.87–1.91), respectively. Although not statistically significant, the risk of follicular lymphoma (HR = 2.12, 95% CI = 0.58, 8.66) and Mature T/NK-cell lymphomas (HR = 2.11, 95% CI = 0.67, 6.66) remained two times higher risk of NHL compared to the non-breast cancer even more than 5 years after the initial diagnosis. Among the Mature T/NK-cell lymphomas, ALCL remained significantly elevated more than 5 years after the initial diagnosis (fully adjusted HR = 10.73; 95% CI = 2.42–47.56). The pattern was similar in women <50 or ≥50 years old (Supplement Table 2).

**Table 3:** Association of incident NHL with incident breast cancer according to the time after diagnosis.

Incidence of non-Hodgkin lymphoma		Non-Hodgkin lymphoma	DLBCL	Follicular lymphoma	Mature T/NK-cell lymphomas	ALCL	Other NHL*
No breast cancer		*Reference*	*Reference*	*Reference*	*Reference*	*Reference*	*Reference*
<1 year after diagnosis	*HR (95% CI)*	2.59 (1.79, 3.74)	2.36 (1.05, 5.29)	7.20 (2.63, 19.70)	3.62 (1.15, 11.40)	–	2.20 (1.34, 3.61)
	*P-value*	<0.01	0.04	<0.01	0.03	–	<0.01
1 to < 3 years after diagnosis	*HR (95% CI)*	1.62 (1.13, 2.31)	0.68 (0.22, 2.12)	4.34 (1.59, 11.89)	3.52 (1.44, 8.63)	9.71 (2.22, 42.51)	1.53 (0.97, 2.41)
	*P-value*	<0.01	0.51	<0.01	<0.01	<0.01	0.07
3 to < 5 years after diagnosis	*HR (95% CI)*	1.41 (0.91, 2.19)	1.19 (0.44, 3.19)	1.48 (0.20, 10.63)	1.90 (0.47, 7.70)	6.87 (0.91, 52.14)	1.43 (0.83, 2.47)
	*P-value*	0.13	0.73	0.70	0.37	0.06	0.20
≥5 years after diagnosis	*HR (95% CI)*	1.29 (0.87, 1.91)	1.05 (0.43, 2.53)	2.12 (0.58, 8.66)	2.11 (0.67, 6.66)	10.73 (2.42, 47.56)	1.22 (0.73, 2.03)
	*P-value*	0.21	0.92	0.30	0.20	<0.02	0.45

ALCL anaplastic large cell lymphoma, DLBCL Diffuse large B-cell lymphoma, NHL non-Hodgkin lymphoma, HR hazard ratio, CI confidence interval, NHL non-Hodgkin lymphoma.

Age as the time scale. Adjusted for body mass index categories (underweight, normal, overweight, obese, and unknown), alcohol consumption (none, moderate, heavy, and unknown), moderate–vigorous physical activity (none, 1–2 times per week, >3 times per week, and unknown), smoking status (never, ever, or unknown), and income percentile (Medicaid, ≤30th, 31st–70th, >70th).

Among the patients who developed breast cancer, the most common combinations of therapies with surgery were chemotherapy and radiotherapy (*N* = 38,541, 45.4%); radiotherapy (*N* = 14,836, 17.5%); and radiotherapy, chemotherapy, and hormone therapy (*N* = 10,970, 12.9%) (Fig. 1). While other treatments were not associated with an increased incidence of NHL, patients who received hormone therapy had a substantially increased risk of all subtypes of NHL compared to patients who did not receive hormone therapy (fully adjusted HR = 2.68; 95% CI = 1.83–3.94) (Table 4). Especially, risk of DLBCL (HR = 2.15; 95% CI = 0.83, 5.55) and NK/T lymphoma (HR = 3.91; 95% CI = 1.31, 11.64) were increased after breast cancer among patients with anti-hormone therapy.

**Table 4:** Association of incident NHL with incident breast cancer according to treatment status.

Type of treatment after breast cancer	Non-Hodgkin lymphoma HR (95% CI) *P*-value	DLBCL HR (95% CI) *P*-value	Follicular lymphoma HR (95% CI) *P*-value	Mature T/NK-cell lymphomas HR (95% CI) *P*-value	ALCLHR (95% CI) *P*-value	Other NHL* HR (95% CI) *P*-value
*Any treatment*						
Incident breast cancer without adjuvant therapy	*Reference*	*Reference*	*Reference*	*Reference*	*Reference*	*Reference*
Incident breast cancer with adjuvant therapy	0.77 (0.41, 1.43) 0.40	0.50 (0.15, 1.74) 0.28	0.37 (0.08, 1.71) 0.20	–	–	0.83 (0.36, 1.92) 0.66
*Chemotherapy*						
Incident breast cancer without chemotherapy	*Reference*	*Reference*	*Reference*	*Reference*	*Reference*	*Reference*
Incident breast cancer with chemotherapy	0.76 (0.51, 1.12) 0.16	0.59 (0.23, 1.48) 0.26	0.40 (0.12, 1.30) 0.13	1.09 (0.34, 3.57) 0.88	0.68 (0.11, 4.13) 0.67	0.83 (0.50, 1.39) 0.49
*Radiation therapy*						
Incident breast cancer without radiation therapy	*Reference*	*Reference*	*Reference*	*Reference*	*Reference*	*Reference*
Incident breast cancer with radiation therapy	0.95 (0.61, 1.48) 0.81	1.17 (0.39, 3.56) 0.78	0.78 (0.21, 2.96) 0.72	0.98 (0.27, 3.58) 0.98	0.42 (0.07, 2.51) 0.34	0.90 (0.51, 1.59) 0.72
*Hormone therapy*						
Incident breast cancer without hormone therapy	*Reference*	*Reference*	*Reference*	*Reference*	*Reference*	*Reference*
Incident breast cancer with hormone therapy	2.68 (1.83, 3.94) < 0.01	2.15 (0.83, 5.55) 0.11	1.26 (0.33, 4.75) 0.73	3.91 (1.31, 11.64) 0.01	4.81 (0.80, 28.89) 0.09	2.98 (1.82, 4.89) < 0.01

HR hazard ratio, CI confidence interval, ALCL anaplastic large cell lymphoma, DLBCL Diffuse large B-cell lymphoma, NHL non-Hodgkin lymphom.

Age as the time scale. Adjusted for body mass index categories (underweight, normal, overweight, obese, and unknown), alcohol consumption (none, moderate, heavy, and unknown), moderate–vigorous physical activity (none, 1–2 times per week, >3 times per week, and unknown), smoking status (never, ever, or unknown), and income percentile (Medicaid, ≤30th, 31st–70th, >70th).

The development of breast cancer was associated with a significantly increased risk of both NHL other than ALCL (fully adjusted HR = 1.57; 95% CI = 1.28–1.93, Fig. 2B and Table 2) and ALCL (fully adjusted HR = 7.46; 95% CI = 2.70–20.59, Fig. 2C and Table 2). Moreover, the risk of ALCL remained significantly elevated more than 5 years after the initial diagnosis (fully adjusted HR = 15.52; 95% CI = 4.42–54.41).

## Discussion

In this large national cohort, breast cancer increased the risk of subsequent development of NHL. The risk of NHL was much higher in patients who were younger than 50 years and patients who received anti-hormone therapy. Especially higher risk of DLBCL was associated with anti-hormone therapy. The increased risk of NHL was evident shortly after cancer development, was highest in the first 2 years after a breast cancer diagnosis, and remained elevated throughout the rest of the follow-up period.

While previous studies have observed an increased incidence of treatment-related leukemia following chemotherapy [18], its association with the incidence of NHL is controversial [12]. In our study, we observed no association between chemotherapy or radiation therapy and NHL incidence. However, hormone therapy was significantly associated with an increased risk of NHL. According to previous literature, estrogen may have protective effects against the risk of NHL [19, 20]. In a population-based case-control study, women with NHL who had taken oral contraceptives or estrogen-containing lactation suppressants demonstrated that estrogen exposure essentially reduced the risk of NHL by half [21]. In contrast, anti-hormone therapy was associated with an increased risk of lymphoma. A tamoxifen trial demonstrated an increased risk of leukemia/lymphoma/myeloma among women randomized to the tamoxifen group (HR = 1.58, 95% CI = 0.86–2.98) [22]. However, the causal relationship between estrogen and NHL is unclear and could vary according to subtype. In this study, we found that the risk of follicular lymphoma and mature T/NK-cell lymphomas was increased after breast cancer. According to a meta-analysis of cohort data evaluating the association of hormone replacement therapy and the risk of NHL, postmenopausal hormone replacement therapy reduced the risks of DLBCL and FL (pooled OR = 0.66, 95% CI 0.54–0.80; pooled OR = 0.82, 95% CI 0.66–1.01, respectively) [17]. The mechanisms by which breast cancer may lead to FL or other subtypes of NHL are uncertain but may involve estrogen-related pathways on the immune system. Sex hormones are known to affect B-cell development, cytokine production, and cytokine receptor expression [23].

In our study, the association between breast cancer and the diagnosis of NHL was significantly stronger in patients younger than 50 years than in those older than 50 years. Young age has been identified as a risk factor for the development of second primary cancers among breast cancer survivors [24, 25]. Furthermore, age < 50 years was associated with a higher risk of second primary cancers [26]. Patients who develop breast cancer at a young age might have more genetic and environmental risk factors, and aggressive treatment for breast cancer might lead to carcinogenesis [27–29]. Thus, further attention is required in young populations. Currently, surveillance is recommended in breast cancer survivors after initial treatment based on their symptoms [30]. Therefore, the development of more comprehensive care strategies is required based on research on the epidemiology, screening, and chemoprevention of second primary cancers [31]. We also found that the patients who received anti-estrogen therapy had a higher risk of NHL, particularly of DLBCL. While FL is associated with estrogenic effects, DLBCL may be more related to a lack of estrogen status. Castration accelerated lymphoma growth in C57BL6 male mice grafted with murine EG7 T cell lymphoma cells. In contrast, inhibition of androgen-to-estrogen conversion by the letrozole induced faster lymphoma growth in mice, suggesting that androgens impact lymphoma growth through their conversion to estrogens [32]. Further studies are necessary to evaluate the impact of hormones on incident NHL.

We also observed a high excess risk of NHL within 3 years of breast cancer diagnosis. These effects might be associated with hormone deprivation due to breast cancer treatment. In contrast, the risk of ALCL remained significantly elevated more than 5 years after the initial diagnosis. As reported by the US and global data as of July 2019, the median age at the time of diagnosis was 53 years (range 27–90 years), and the median time from the last breast implantation to an ALCL diagnosis was 8 years (range 0–34 years) [33]. In a report of six cases of BIA-ALCL in Germany, most cases manifested 7–10 years after implantation [34]. As the number of breast cancer survivors undergoing breast plastic surgery increases by five-fold each year [35], the cumulative incidence of BIA-ALCL is rapidly increasing; [36] thus, the National Comprehensive Cancer Network published guidelines on BIA-ALCL in 2019 [37]. According to these guidelines, when diagnosed early, BIA-ALCL is commonly indolent and slow-growing with an excellent prognosis. Therefore, appropriate surveillance should be performed to ensure timely diagnosis and treatment. Patients with symptomatic periprosthetic effusions occurring more than 1 year after implantation should be tested for BIA-ALCL [37].

Our study has several limitations. First, data on exposure and outcomes were extracted from claims data. However, the identification of cancer cases based on claims is considered reliable in Korea, as cancer codes are reviewed by the NHIS and have implications for additional patient benefits. While these data are subject to errors, claims data for the clinical outcomes evaluated in this study are highly specific and have been widely used in population studies. Second, the NHIS claims data do not contain information on laboratory, imaging, pathology, family history, or reproductive profile; we did not have information on cancer stage, type of hormone, and germline mutations in the breast tumors. Further studies with detailed clinical data are necessary to confirm the study findings. Third, there are services and treatments that are not covered by national health insurance, including preventative surgery, over-the-counter drugs, and cosmetic surgery. Thus, we could not determine whether the participants had received breast implants. Since 2015, the national health insurance in Korea has covered breast reconstruction; thus, studies are needed after the accumulation of sufficient follow-up data to observe cancer outcomes. In addition, we were not able to adjust for the potential confounding effects of breast implants or birth control pill use. However, when we used data from the baseline health screening exam, the proportion of women who took birth control pills among those without and with breast cancer was 5.5 and 4.7%. In addition, the proportion of hormone replacement therapy at the baseline health screening exam was similar among women without (10%) and with (9.7%) breast cancer. It is thus unlikely that the use of estrogen-based contraceptives or hormone replacement therapy can explain the differences between the groups. Fifths, patients who develop breast cancer have more frequent contact with the health care system, which may induce surveillance bias. Finally, although we had sufficient statistical power to observe the incidence of NHL in breast cancer patients, a longer follow-up duration may be needed given the incidence of various subtypes of NHL. However, this study’s strength was its nationwide cohort study design that included a relatively large number of NHL cases and consideration of the types of cancer treatments and lifestyle variables that could affect the occurrence of a second malignancy. Finally, the association of breast cancer with overall NHL may depend on the breast cancer treatment or the mix of subtypes of NHL, which may be different in different populations [38]. One of the key characteristics of breast cancer in Korea is the higher incidence of young-age breast cancer than in Western countries [39]. Related to the difference in age distribution, the pattern of treatment for breast cancer is also different than in Western countries. Additionally, the type of NHL also differs with respect to western. Compared to Western countries, Korea has a higher frequency of follicular lymphoma and NK/T cell lymphomas which are relatively more strongly associated with incident breast cancer [40]. Therefore, our findings may not be generalizable to other settings with different incidence rates for different types of NHL. Regardless of the limitations, this study has several strengths. The study used a national dataset that included a relatively large number of NHL cases. We also had information on the types of cancer treatment. In addition, we used health screening examination data and could include important lifestyle variables that could affect the occurrence of a second malignancy such as smoking drinking, physical activity, and BMI.

In conclusion, breast cancer development was associated with an increased risk of NHL development. In particular, the risk of NHL was higher in patients receiving hormone therapy and in younger patients. In addition, the excess risk of NHL was higher within 3 years after breast cancer diagnosis. Surveillance guidelines should be developed to screen for NHL in patients with breast cancer, especially those diagnosed at a younger age and who received hormone therapy.

## Supplementary information

Reproducibility checklist

UpSet plot of treatment combinations in patients with breast cancer

Factors associated with the incidence of Non-Hodgkin lymphoma
