# Supplementary material for: Risk of non-Hodgkin lymphoma in breast cancer survivors: a nationwide cohort study
Source: Blood Cancer J. 2021 Dec 14;11(12):200. doi: 10.1038/s41408-021-00595-0 (PMC8671407; doi:10.1038/s41408-021-00595-0)

**eFigure 1.** **UpSet plot of treatment combinations in patients with breast cancer**

The vertical bars reflect the numbers of participants receiving a specific combination of treatments, identified by the dots below each bar. For instance, the first vertical bar indicates that 38,541 participants were treated with surgery plus radiation therapy plus chemotherapy but not hormone therapy. The second vertical bar indicates that 14,836 participants were treated with surgery plus radiation therapy but not chemotherapy or hormone therapy. The horizontal bars reflect the numbers of participants who received each treatment across all treatment combinations.

CTx, chemotherapy; HTx, hormone therapy; RTx, radiation therapy


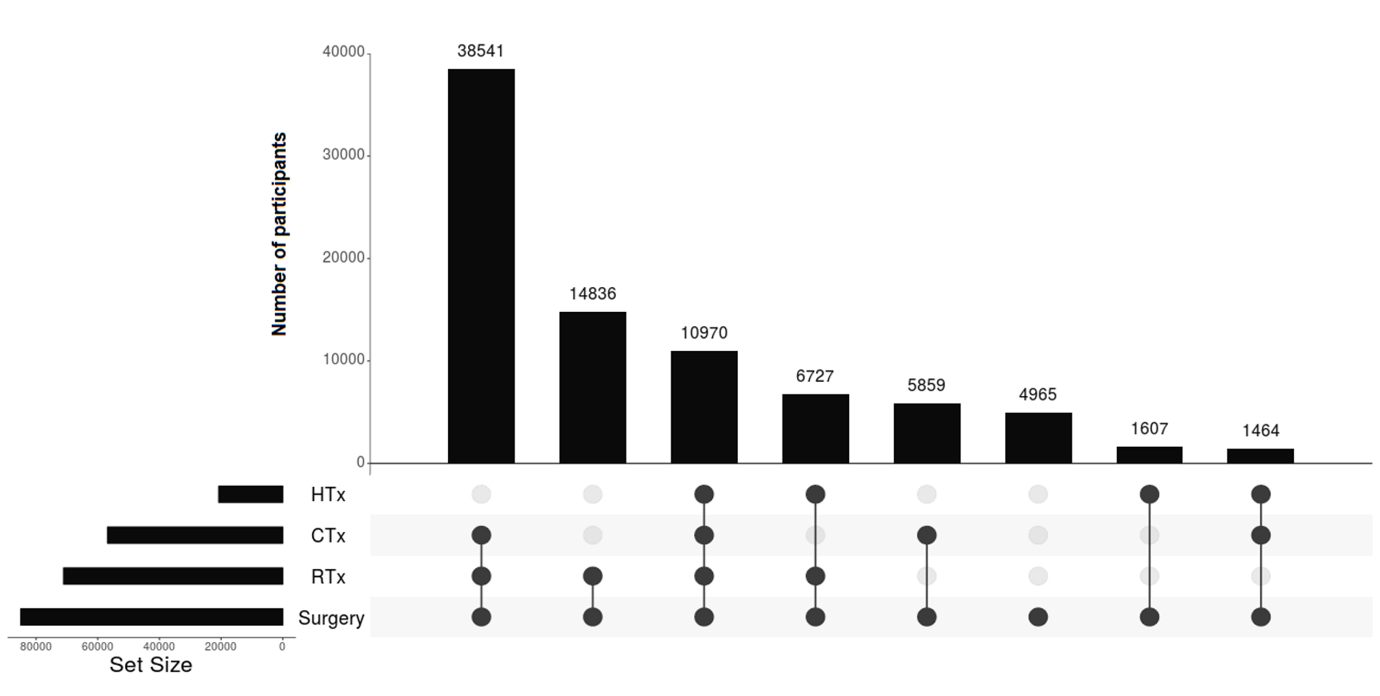

Supplement: Supplementary file 2 — UpSet plot of treatment combinations in patients with breast cancer [file 41408_2021_595_MOESM2_ESM.docx]
