# Supplementary material for: Risk of non-Hodgkin lymphoma in breast cancer survivors: a nationwide cohort study
Source: Blood Cancer J. 2021 Dec 14;11(12):200. doi: 10.1038/s41408-021-00595-0 (PMC8671407; doi:10.1038/s41408-021-00595-0)
Supplement: Supplementary file 3 — Factors associated with the incidence of Non-Hodgkin lymphoma [file 41408_2021_595_MOESM3_ESM.docx]

**Supplement table 1. Factors associated with incidence of Non-Hodgkin lymphoma**

| **Characteristics** | **NHL**  **Adjusted HR (95% CI)**  **P value** | **DLBCL**  **Adjusted HR (95% CI)**  **P value** | **Follicular lymphoma**  **Adjusted HR (95% CI)**  **P value** | **Mature T/NK-cell lymphomas**  **Adjusted HR (95% CI)**  **P value** | **Other NHL***  **Adjusted HR (95% CI)**  **P value** |
| --- | --- | --- | --- | --- | --- |
| **Age at menarche, years** |  |  |  |  |  |
| <14 | 0.95 (0.64, 1.41)  0.80 | 0.77 (0.36, 1.67)  0.51 | 0.95 (0.64, 1.41)  0.80 | 0.80 (0.19, 3.33)  0.76 | 1.10 (0.65, 1.84)  0.73 |
| 14-16 | 0.96 (0.65, 1.42)  0.84 | 0.94 (0.44, 1.99)  0.86 | 0.96 (0.65, 1.42)  0.84 | 0.97 (0.24, 3.97)  0.97 | 1.02 (0.61, 1.70)  0.95 |
| ≥ 17 | Reference | Reference | Reference | Reference | Reference |
| **Menopause status** |  |  |  |  |  |
| Pre-menopause | Reference | Reference | Reference | Reference | Reference |
| Post-menopause | 0.94 (0.80, 1.10)  0.43 | 0.73 (0.51, 1.04)  0.08 | 0.94 (0.80, 1.10)  0.43 | 0.73 (0.42, 1.28)  0.27 | 1.05 (0.86, 1.70)  0.63 |
| **BMI, kg/m^2^** |  |  |  |  |  |
| Underweight (< 18) | Reference | Reference | Reference | Reference | Reference |
| Normal (18 to < 23) | 1.21 (0.86, 1.71)  0.27 | 1.66 (0.88, 4.06)  0.27 | 1.21 (0.86, 1.71)  0.27 | 0.84 (0.80, 2.56)  0.74 | 1.30 (0.85, 2.01)  0.23 |
| Overweight (23 to < 25) | 1.27 (0.89, 1.80)  0.18 | 1.59 (0.64, 3.94)  0.32 | 1.27 (0.89, 1.80)  0.18 | 0.78 (0.27, 2.28)  0.65 | 1.38 (0.89, 2.15)  0.15 |
| Obese (≥ 25) | 1.33 (0.94, 1.88)  0.11 | 1.76 (0.72, 4.32)  0.22 | 1.33 (0.94, 1.88)  0.11 | 0.95 (0.33, 2.73)  0.93 | 1.42 (0.92, 2.20)  0.12 |
| Missing data |  |  |  |  |  |
| **Alcohol consumption** |  |  |  |  |  |
| None | Reference | Reference | Reference | Reference | Reference |
| Moderate (1–19 g/day) | 0.91 (0.80, 1.04)  0.18 | 0.84 (0.62, 1.13)  0.24 | 0.91 (0.80, 1.04)  0.18 | 1.23 (0.80, 1.89)  0.35 | 0.88 (0.75, 1.04)  0.14 |
| Heavy (≥ 20 g/day) | 0.83 (0.52, 1.33)  0.44 | 0.76 (0.24, 2.40)  0.64 | 0.83 (0.52, 1.33)  0.44 | 0.64 (0.09, 4.68)  0.66 | 0.93 (0.54, 1.60)  0.80 |
| **Smoking status** |  |  |  |  |  |
| Never smoker | Reference | Reference | Reference | Reference | Reference |
| Ever smoker | 1.19 (0.94, 1.50)  0.15 | 0.87 (0.48, 1.57)  0.65 | 1.19 (0.94, 1.50)  0.15 | 1.08 (0.47, 2.50)  0.86 | 1.30 (0.99, 1.72)  0.06 |
| **Moderate–vigorous physical activity** |  |  |  |  |  |
| None | Reference | Reference | Reference | Reference | Reference |
| 1–2 times per week | 1.00 (0.88, 1.14)  0.99 | 0.98 (0.73, 1.31)  0.88 | 1.00 (0.88, 1.14)  0.99 | 0.96 (0.60, 1.56)  0.88 | 1.01 (0.86, 1.19)  0.91 |
| ≥ 3 times per week | 0.93 (0.81, 1.06)  0.28 | 0.88 (0.66, 1.17)  0.38 | 0.93 (0.81, 1.06)  0.28 | 1.02 (0.64, 1.62)  0.95 | 0.91 (0.77, 1.07)  0.26 |
| **Income percentile** |  |  |  |  |  |
| Medical Aid | Reference | Reference | Reference | Reference | Reference |
| ≤ 30^th^ | 0.86 (0.58, 1.28)  0.46 | 0.57 (0.28, 1.18)  0.13 | 0.86 (0.58, 1.28)  0.46 | 0.84 (0.20, 3.56)  0.81 | 0.99 (0.59, 1.66)  0.97 |
| 31^st^–70^th^ | 0.82 (0.55, 1.22)  0.32 | 0.65 (0.32, 1.33)  0.24 | 0.82 (0.55, 1.22)  0.32 | 0.77 (0.18, 3.19)  0.71 | 0.90 (0.53, 1.50)  0.68 |
| >70^th^ | 0.97 (0.66, 1.44)  0.90 | 0.72 (0.35, 1.47)  0.37 | 0.97 (0.66, 1.44)  0.90 | 1.09 (0.26, 4.46)  0.91 | 1.07 (0.64, 1.80)  0.79 |

Age as time scale. Adjusted for incident breast cancer, age at menarche, years ( <14, 14-16, ≥ 17 and unknown), menopause status (pre-, post-, and unknown), body mass index categories (underweight, normal, overweight, obese, and unknown), alcohol consumption (none, moderate, heavy, and unknown), moderate–vigorous physical activity (none, 1–2 times per week, > 3 times per week, and unknown), smoking status (never, ever, and unknown), and income percentile (Medicaid, ≤ 30th, 31^st^–70^th^, > 70th).

**Supplement Table 2.** Association of Incident NHL with Incident Breast Cancer According to the Time after Diagnosis

| **Incidence of non-Hodgkin lymphoma** | | **Non-Hodgkin lymphoma** | **DLBCL** | **Follicular lymphoma** | **Mature T/NK-cell lymphomas** | **ALCL** | **Other NHL*** |
| --- | --- | --- | --- | --- | --- | --- | --- |
| **Age < 50 years** | |  |  |  |  |  |  |
| **No breast cancer** | | *Reference* | *Reference* | *Reference* | *Reference* | *Reference* | *Reference* |
| **< 1 year after diagnosis** | **HR (95% CI)** | 3.05  (1.57, 5.91) | 1.99  (0.28, 14.35) | 14.67  (3.38, 63.73) | 4.21  (0.57, 30.92) | - | 2.41  (0.99, 5.84) |
|  | **P value** | < 0.01 | 0.50 | < 0.01 | 0.16 | - | 0.05 |
| **1 to < 3 years after diagnosis** | **HR (95% CI)** | 2.55  (1.40, 4.65) | 1.32  (0.18, 9.55) | 5.23  (0.69, 39.45) | 2.81  (0.38, 20.66) | 25.03  (2.59, 241.83) | 2.66  (1.31, 5.37) |
|  | **P value** | < 0.01 | 0.78 | 0.11 | 0.31 | < 0.01 | < 0.01 |
| **3 to < 5 years after diagnosis** | **HR (95% CI)** | 4.22  (2.25, 7.46) | 2.30  (0.32, 16.63) | 10.20  (1.34, 77.37) | 4.85  (0.66, 35.79) | 42.89  (4.39, 418.56) | 4.28  (2.01, 9.08) |
|  | **P value** | 0.34 | 0.41 | 0.03 | 0.72 | < 0.01 | < 0.01 |
| **≥ 5 years after diagnosis** | **HR (95% CI)** | 1.75  (0.56, 5.46) | - | 15.75  (2.06, 120.48) | - | - | 1.69  (0.42, 6.80) |
|  | **P value** | 0.34 |  | < 0.01 |  |  | 0.46 |
| **Age ≥ 50 years** |  |  |  |  |  |  |  |
| **No breast cancer** | | *Reference* | *Reference* | *Reference* | *Reference* | *Reference* | *Reference* |
| **< 1 year after diagnosis** | **HR (95% CI)** | 2.43  (1.56, 3.78) | 2.45  (1.01, 5.94) | 4.80  (1.17, 19.66) | 3.38  (0.83, 13.70) | - | 2.13  (1.17, 3.86) |
|  | **P value** | 0.01 | 0.05 | 0.03 | 0.09 | - | 0.01 |
| **1 to < 3 years after diagnosis** | **HR (95% CI)** | 1.35  (0.87, 2.10) | 0.55  (0.14, 2.20) | 4.10  (1.28, 13.09) | 3.76  (1.38, 10.26) | 6.01  (0.78, 46.27) | 1.17  (0.65, 2.13) |
|  | **P value** | 0.19 | 0.40 | 0.02 | 0.09 | < 0.01 | 0.60 |
| **3 to < 5 years after diagnosis** | **HR (95% CI)** | 0.84  (0.45, 1.57) | 1.02  (0.33, 3.19) | - | 1.18  (0.16, 8.48) | 11.10  (2.47, 49.88) | 0.80  (0.36, 1.79) |
|  | **P value** | 0.59 | 0.67 |  | 0.87 | < 0.01 | 0.59 |
| **≥ 5 years after diagnosis** | **HR (95% CI)** | 1.23  (0.81, 1.88) | 1.12  (0.46, 2.71) | 1.10  (0.15, 7.93) | 2.35  (0.74, 7.46) |  | 1.16  (0.67, 2.01) |
|  | **P value** | 0.33 | 0.80 | 0.93 | 0.15 |  | 0.59 |

ALCL, anaplastic large cell lymphoma; DLBCL, Diffuse large B-cell lymphoma; NHL, non-Hodgkin lymphoma; HR, hazard ratio; CI, confidence interval; NHL, non-Hodgkin lymphoma

Age as time scale. Adjusted for body mass index categories (underweight, normal, overweight, obese, and unknown), alcohol consumption (none, moderate, heavy, and unknown), moderate–vigorous physical activity (none, 1–2 times per week, > 3 times per week, and unknown), smoking status (never, ever, or unknown), and income percentile (Medicaid, ≤ 30th, 31st–70th, > 70th)
